# Injectable Magnetic-Responsive Short-Peptide Supramolecular Hydrogels: Ex Vivo and In Vivo Evaluation

**DOI:** 10.1021/acsami.1c13972

**Published:** 2021-10-14

**Authors:** Mari C. Mañas-Torres, Cristina Gila-Vilchez, Francisco J. Vazquez-Perez, Pavel Kuzhir, David Momier, Jean-Claude Scimeca, Arnaud Borderie, Marianne Goracci, Fanny Burel-Vandenbos, Cristina Blanco-Elices, Ismael A. Rodriguez, Miguel Alaminos, Luis Álvarez de Cienfuegos, Modesto T. Lopez-Lopez

**Affiliations:** † Universidad de Granada, Departamento de Química Orgánica, Facultad de Ciencias, Unidad de Excelencia de Química Aplicada a Biomedicina y Medioambiente, 18071 Granada, Spain; ‡ Universidad de Granada, Departamento de Física Aplicada, Facultad de Ciencias, 18071 Granada, Spain; § Instituto de Investigación Biosanitaria (ibs.GRANADA), 18012 Granada, Spain; ∥ Université Côte d’Azur, CNRS UMR 7010, Institute of Physics of Nice, Parc Valrose, 06108 Nice, France; ⊥ Université Côte d’Azur, CNRS UMR 7277, Institute of Biology Valrose, 06107 Nice, France; # Université Côte d’Azur, Department of Pathology, CHU Nice, 06107 Nice, France; ∇ University of Granada, Department of Histology and Tissue Engineering Group, Faculty of Medicine, 18071 Granada, Spain; ○ Department of Histology, Faculty of Dentistry, National University of Cordoba, 5000 Cordoba, Argentina

**Keywords:** peptides, hybrid hydrogels, biomaterials, magnetic nanoparticles, self-assembly, tissue engineering, regenerative medicine

## Abstract

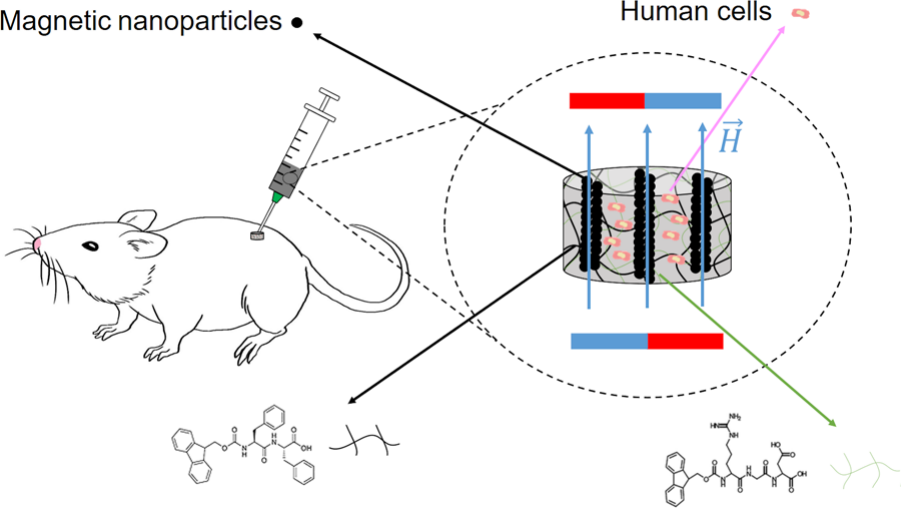

The inclusion of magnetic nanoparticles (MNP) in a hydrogel matrix to produce magnetic hydrogels has broadened the scope of these materials in biomedical research. Embedded MNP offer the possibility to modulate the physical properties of the hydrogel remotely and on demand by applying an external magnetic field. Moreover, they enable permanent changes in the mechanical properties of the hydrogel, as well as alterations in the micro- and macroporosity of its three-dimensional (3D) structure, with the associated potential to induce anisotropy. In this work, the behavior of biocompatible and biodegradable hydrogels made with Fmoc-diphenylalanine (Fmoc-FF) (Fmoc = fluorenylmethoxycarbonyl) and Fmoc–arginine–glycine–aspartic acid (Fmoc-RGD) short peptides to which MNP were incorporated was studied in detail with physicochemical, mechanical, and biological methods. The resulting hybrid hydrogels showed enhance mechanical properties and withstood injection without phase disruption. In mice, the hydrogels showed faster and improved self-healing properties compared to their nonmagnetic counterparts. Thanks to these superior physical properties and stability during culture, they can be used as 3D scaffolds for cell growth. Additionally, magnetic short-peptide hydrogels showed good biocompatibility and the absence of toxicity, which together with their enhanced mechanical stability and excellent injectability make them ideal biomaterials for in vivo biomedical applications with minimally invasive surgery. This study presents a new approach to improving the physical and mechanical properties of supramolecular hydrogels by incorporating MNP, which confer structural reinforcement and stability, remote actuation by magnetic fields, and better injectability. Our approach is a potential catalyst for expanding the biomedical applications of supramolecular short-peptide hydrogels.

## Introduction

Injectable hydrogels are useful materials with advanced biomedical applications in the fields of tissue engineering and drug delivery (including macromolecules and cells) and in surgery as void-fillers, bioadhesives, or antiadhesives.^1−3^ These hydrogels show potential for use in therapy with minimally invasive procedures requiring only a needle and therefore with reduced surgical time, pain, and complications. For clinical purposes, these materials must meet high standards involving specific technical requirements to successfully perform their designated functions.^4−6^ Moreover, they must be completely biocompatible and biodegradable because they are intended for use inside the human body. Biodegradability inside the body is a key issue, because drug delivery and cell adhesion or regeneration are controlled by and can be affected by the rate of hydrogel degradation; accordingly, the physiological and therapeutic effects are also modulated by this process.^7^ The physical properties of these materials must be well controlled, remain within specific parameters to ensure that the materials are fluid enough to be injected, and be rigid or compact enough to withstand and/or transport cells or drugs. In addition, they must remain temporarily localized within a specific tissue in order to exert a therapeutic effect within a specified period.^8^ Biocompatibility and physical properties both depend on the chemical strategy used to develop these systems.^7^

To achieve biocompatibility, hydrogels have been developed mainly with materials from natural sources such as alginate, agarose, collagen, gelatin, silk fibroin, hyaluronic acid, chitosan, cellulose, and starch, alone or in combination with other compounds.^9^ Because injectable hydrogels need to be sufficiently fluid to flow through a syringe needle, the most common strategy to ensure injectability is to inject a pregel mixture or a free-flowing solution that jellifies slowly^1^ or whose gelation can be triggered under physiological conditions by enzymes, salts, temperature changes, or other stimuli.^8^ However, these in situ gelling hydrogels may have some drawbacks since, being liquid, their contents can leak into the body; moreover, the gelling process, as well as the final biomechanical properties, can be affected by in vivo conditions. Alternatively, certain hydrogels present the interesting property of flowing under an applied stress (shear-thinning) and easily recover their original stiffness after removal of the stress (self-healing) without the intervention of any external stimulus.^8^ Shear-thinning hydrogels rely on reversible physical cross-links and include hydrogels made with self-assembling peptides, in particular, β-hairpin peptides, which were studied in detail by Pochan and Schneider,^10−12^ among others.^7,8,13^ Because the latter strategy involves the injection of pre-existing hydrogels, their mechanical and biological properties can be well characterized and studied. Moreover, the recovery of mechanical properties may be faster and less affected by the environment than in gels triggered by external conditions. The hydrogel matrix can also be used to carry and deliver sensitive cargos such as human cells. Consequently, these hydrogels have been considered excellent candidates for cell therapy.^10,12^

Other groups of self-assembling peptides that are able to form physical hydrogels are based on amphiphilic dipeptides,^14,15^ in particular, those containing a fluorenylmethyloxycarbonyl (Fmoc) or naphthyl (Nap) group.^16−19^ These peptides are easily accessible and economically competitive and have therefore been used in many important biotechnological applications,^20−22^ including three-dimensional (3D) scaffolds for cell culture, since they show self-healing under specific conditions.^23−27^ Nevertheless, the injectability of these gels has been less explored. In the particular case of Fmoc-diphenylalanine (Fmoc-FF), it was shown that this gel undergoes phase separation during injection.^28^ In this connection, a strategy to improve the properties of some gels consists of developing composite or hybrid hydrogels made with different organic or organic + inorganic materials.^2^ This strategy has also been applied to Fmoc-dipeptide gels with the aim of improving their mechanical properties.^21,29−31^ Recently, Aviv et al. showed that the combination of Fmoc-FF and hyaluronic acid gives rise to composite hydrogels with enhanced mechanical properties that, at specified ratios, can be injected due to the shear-thinning process, making these gels excellent vehicles for drug delivery applications.^28^ Analogously, Li et al. developed an injectable hydrogel for tissue engineering based on the combination of Nap-FF and silk fibroin.^32^

In a previous work, we showed that the mechanical properties of Fmoc-FF hydrogels can be significantly enhanced by the incorporation of magnetic nanoparticles (MNP) while the microporosity of the gel remains unaltered. Moreover, the incorporation of MNP makes it possible to exert control over the mechanical properties remotely by applying an external magnetic field.^33^ It was recently shown that magnetic hydrogels (hydrogels in which magnetic micro- or nanoparticles have been incorporated) offer specific advantages when designed for biomedical applications such as drug delivery and tissue engineering.^34^ These advantages reflect the greater degree of controllability that magnetic hydrogels can offer. Moreover, the mechanical or physical properties can be controlled remotely—a feature that makes these materials compatible with in vivo applications. In this connection, magnetic hydrogels have been used as pulsatile drug delivery vehicles^35^ and in tissue engineering for magnetomechanical stimulation of different cell lines,^36,37^ among other applications.^38−41^

**Figure 1:**
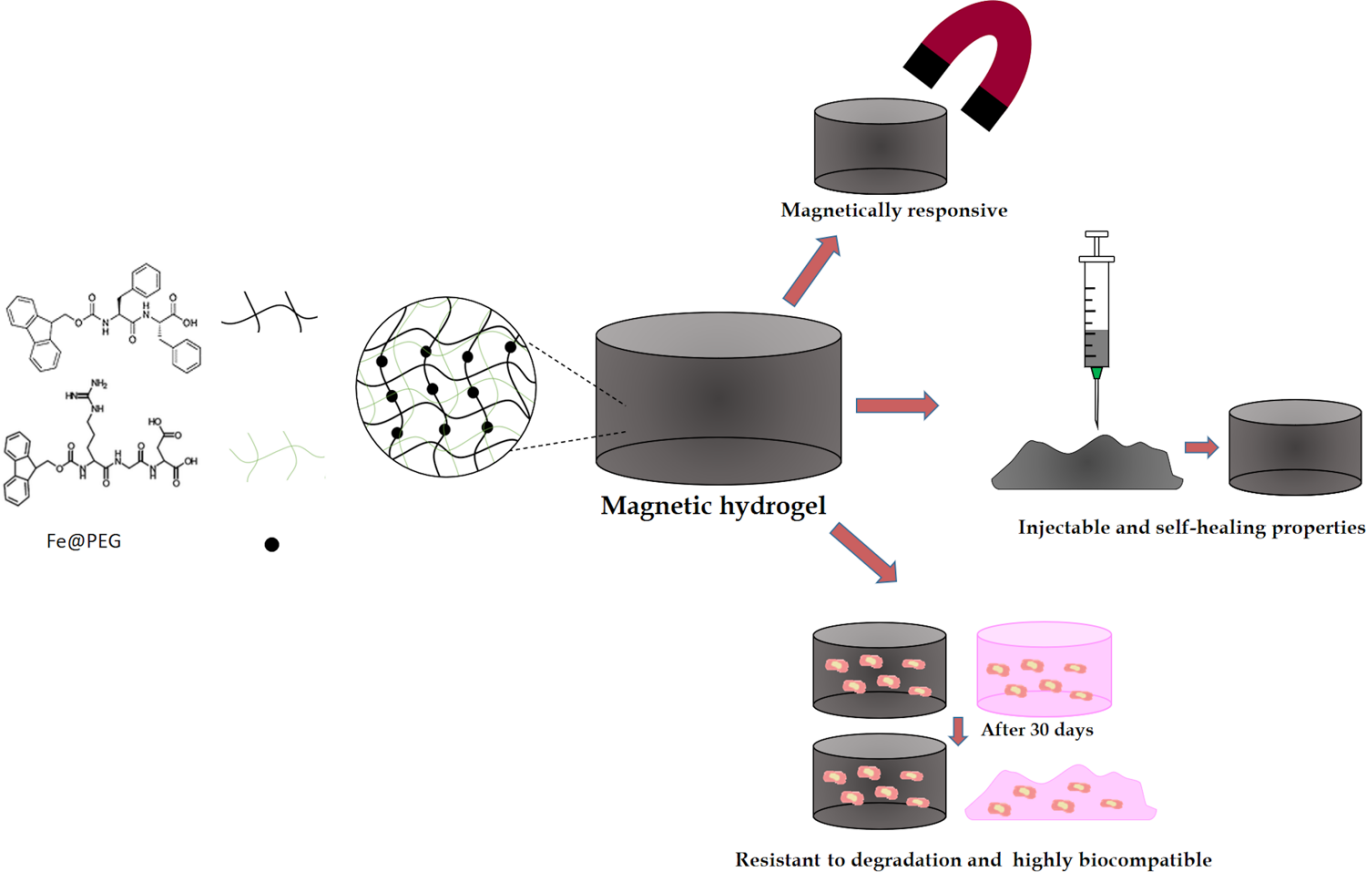
Schematic of the properties of short-peptide supramolecular magnetic hydrogels.

In the present work, we developed a new type of injectable hydrogel that combines within a single system the advantages of injectability through a shear-thinning process with the capacity for remote modulation of its mechanical properties by applying an external magnetic field (Figure 1). This system was obtained by combining Fmoc-FF, Fmoc-RGD (Fmoc–arginine–glycine–aspartic acid), and MNP and has the following unique features: (1) high biocompatibility and biodegradability ex vivo and in vivo; (2) high mechanical stiffness (*G*′ ≥ 20 KPa), making it suitable as a scaffold for cell growth; (3) resistance to degradation with time (scaffolds for cell growth remain unaltered after 30 days); (4) injectability with improved shear-thinning and self-healing properties; and (5) responsiveness to an external magnetic field. These hydrogels can thus be localized in a specific site or can be moved after injection without losing their phase or composition and can therefore be used to translocate the gel or gel cargo to a particular site subcutaneously.

These new systems were tested in vitro as 3D scaffolds for osteoblast growth and as delivery vehicles for injected fibroblasts. In addition, the injectability, toxicity, and persistence of these hydrogels were evaluated in vivo by subcutaneous injection in mice.

## Results and Discussion

### Physicochemical Characterization of Hydrogels

**Figure 2:**
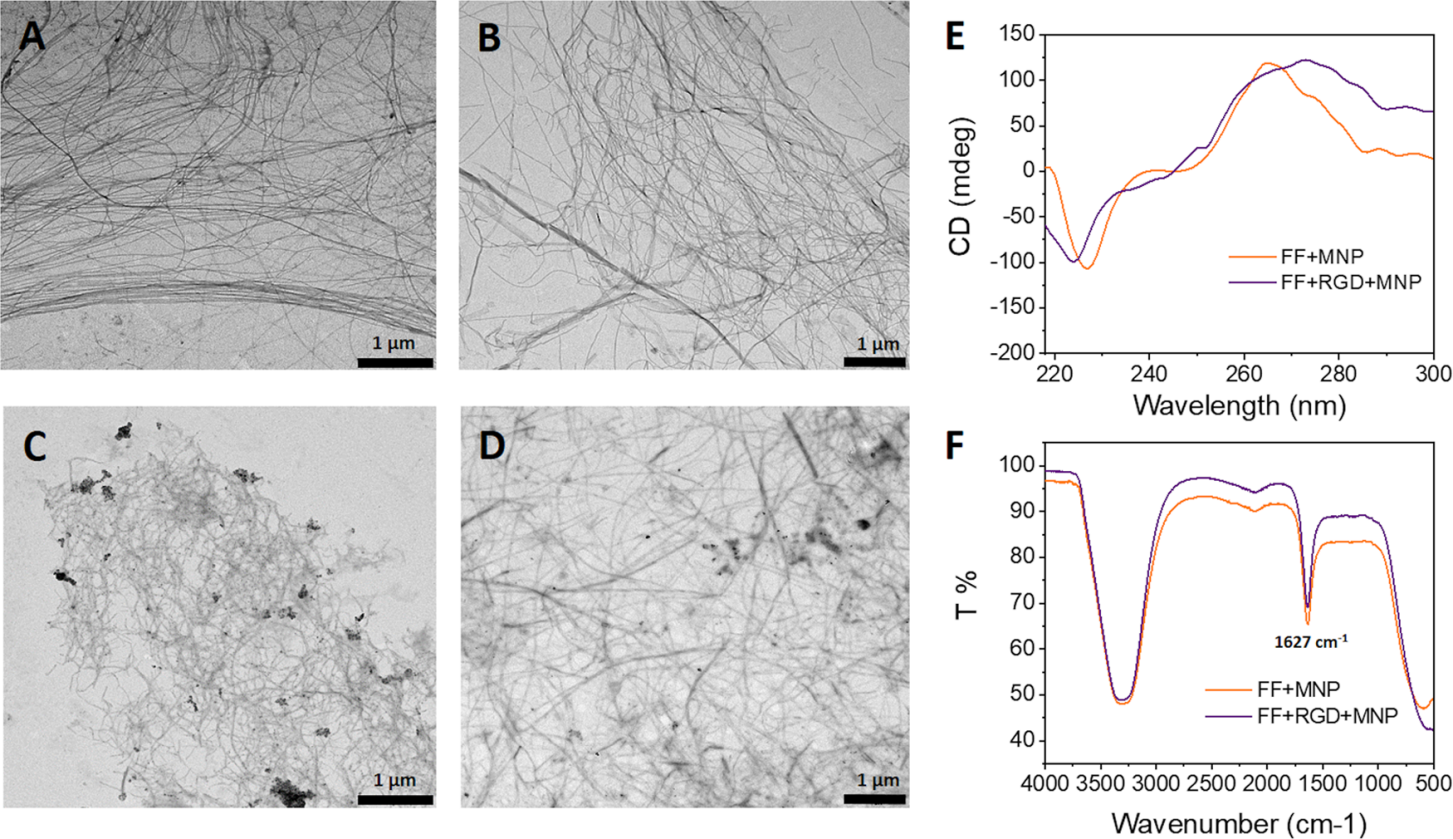
Representative TEM images and CD and FTIR spectra of the hydrogels: (A) Fmoc-FF, (B) Fmoc-FF/Fmoc-RGD, (C) Fmoc-FF + MNP, (D) Fmoc-FF/Fmoc-RGD + MNP, (E) CD of Fmoc-FF + MNP and Fmoc-FF/Fmoc-RGD + MNP, and (F) FTIR of Fmoc-FF + MNP and Fmoc-FF/Fmoc-RGD + MNP.

The morphologies of fibers in Fmoc-FF peptide hydrogels (hereafter, Fmoc-FF = FF samples) and FF/Fmoc-RGD hydrogels (hereafter, Fmoc-RGD = RGD samples) (7:3 ratio) were studied by TEM for systems with and without MNP. Previous TEM studies of FF peptide and FF/RGD mixture fibers have shown the presence of long fibers and ribbons in diameters ranging from 10 to 150 nm and several micrometers in length.^25,42,43^ Ulijn et al. have shown that the morphology of FF/RGD mixture fibers remains similar to those of FF when the amount of RGD is equal or lower than 30 M%, which is the case in the present work.^25^ As seen in Figure 2, TEM images of FF peptide (Figure 2A) show fibers with a length-to-diameter ratio similar to previously reported samples and similar to those shown in Figure 2B corresponding to the FF/RGD mixture, in agreement with the results reported by Ulijn et al.^25^ The incorporation of MNP in both cases (Figure 2C,D) did not appreciably modify fiber morphology when MNP were included in the fiber structure as we have previously reported.^33^

The preservation of fiber morphology in the presence of MNP was also confirmed by analysis of the peptide’s secondary structure within the fibers by CD and FTIR. The CD spectra of FF and FF/RGD showed a broad band near 280 nm corresponding to transitions of the fluorenyl groups and an intense negative peak near 220 nm suggesting a β-sheet-like peptide secondary structure as previously reported^25^ (Figure S1). The similarity of the two spectra confirmed that the arrangement within the peptide mixture gave rise to packaging similar to that of FF alone, which was consistent with TEM images. The CD spectra of the hybrid hydrogels with MNP confirmed that the presence of MNP did not modify the secondary structure of the peptide fibers (Figure 2E). The FTIR spectra also showed compatible results: All samples with (Figure 2F) and without MNP (Figure S1) produced an intense band in the amide I region at 1627 cm^–1^ attributable to formation of β-sheet structures.

### Mechanical Evaluation of Hydrogels

**Figure 3:**
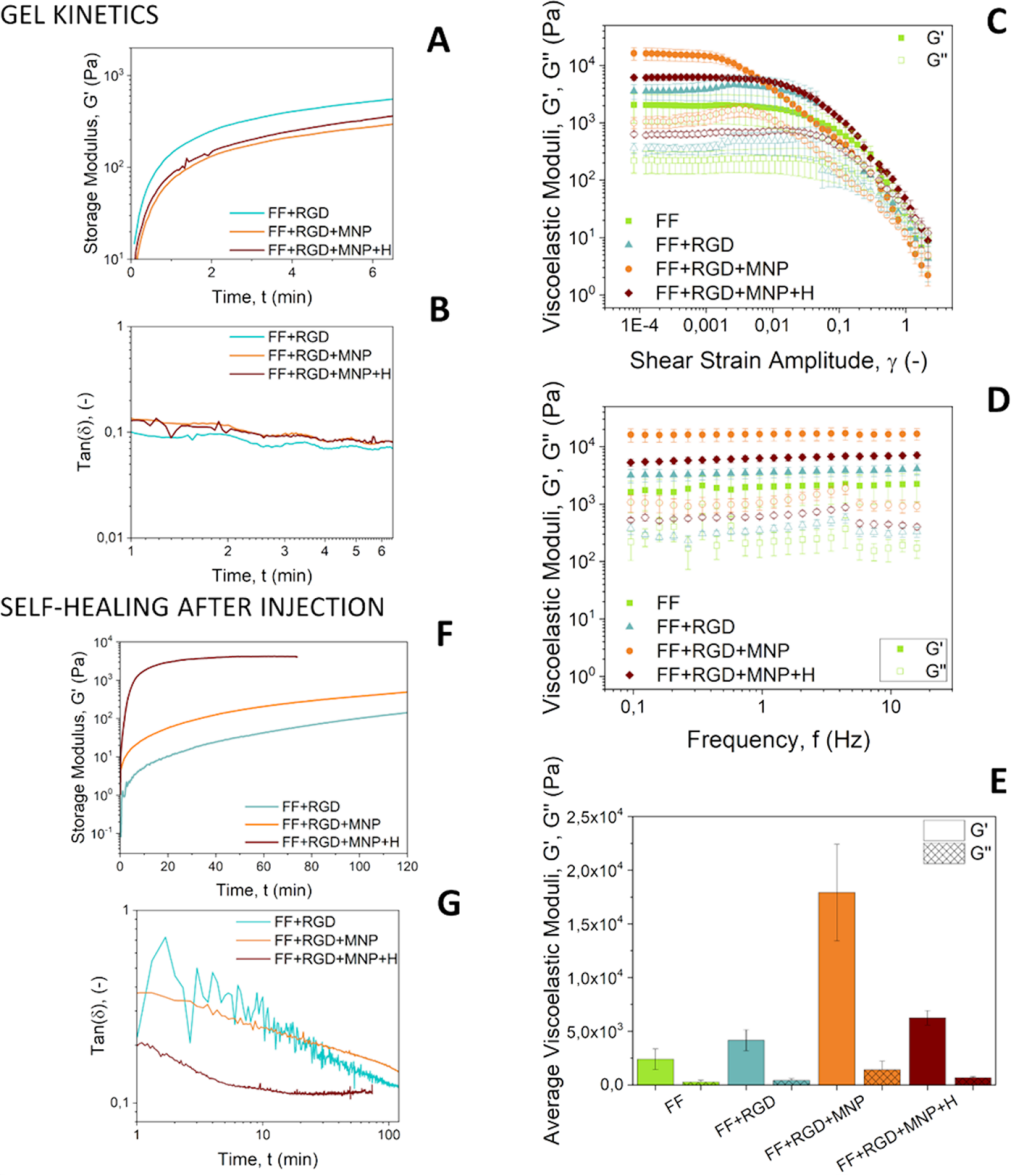
Mechanical response of the hydrogels. Representative curves for storage modulus (A) and loss tangent (B) as a function of time during gelation starting from mixtures of pregel reagents. In amplitude sweeps (C) and frequency sweeps (D) for completely gelled hydrogels, the data represent mean values ± standard errors of at least three independent measurements in different hydrogels for each experimental condition. Mean values ± standard errors (for three different repeats and three different hydrogels, i.e., nine values per experimental condition) of viscoelastic moduli within the LVR (E) for completely gelled hydrogels. Representative curves of the evolution of the storage modulus (F) and loss tangent (G) during a self-healing test after injection of the hydrogels through a syringe.

We first studied the gelation kinetics of our hydrogels by monitoring the evolution of the storage (*G*′) and loss (*G*″) moduli as a function of time, starting from the mixtures of the reagents. As observed, *G*′ increased steeply with time from the very beginning of the test and continued to increase less steeply after a few minutes (Figure 3A), which may be ascribed to rapid, deep gel formation. Indeed, as shown from the loss tangent curves tan(δ) = *G*″/*G*′ vs time during gelation (Figure 3B), the mixtures presented gel-like features from the very beginning of measurements. It should be noted that 0.1 < tan(δ) < 1 is typical of weak gels, whereas strong gels present values of tan(δ) < 0.1. Therefore, our samples demonstrated borderline behavior between weak and strong gels. Regarding differences between samples, they all showed similar gelation kinetics. We also characterized the mechanical properties of hydrogels under oscillatory shear after complete gelation. Amplitude sweeps (Figure 3C) disclosed two identifiable regions: the linear viscoelastic region (LVR) characterized by *G*′ and *G*″ values almost independent of shear strain amplitude and with *G*′ > *G*″ as expected for gel-like materials. At higher values of shear strain amplitude, *G*″ increased steadily up to its maximum value, known as the yield point, while *G*′ decreased steadily. These changes marked the onset of the second region referred to as the nonlinear viscoelastic region (NLVR). Within the NLVR, the viscoelastic moduli decreased markedly in relation to destruction of the gel-like network, and eventually, transition to a liquid-like behavior (*G*′ < *G*″) occurred. The mechanical spectra of samples, i.e., the frequency sweeps for a constant shear strain amplitude (γ_0_ = 0.001) within the LVR (Figure 3D), demonstrated almost frequency-independent trends for *G*′, typical of densely cross-linked gels. In contrast, *G*″ showed a weak tendency to increase up to a frequency of 4 Hz and decreased thereafter. Comparison of the viscoelastic moduli for different samples (Figure 3E) showed that FF samples produced the weakest response (smallest values of *G*′ and *G*″ within the LVR) and the addition of RGD resulted in a slight enhancement in strength, whereas the addition of MNP markedly enhanced strength by inducing an almost 7-fold increase in *G*′ and *G*″. Mechanical strengthening of hydrogels induced by the inclusion of solid particles in the formulation has been widely reported for polymer gels and has been found to be a result of changes in cross-linking or in the microstructure of the 3D polymer network, simply a direct result of embedment of solid inclusions, or a combination of all these effects.^33,44−46^

Application of a magnetic field during gelation (FF + RGD + MNP + H sample) resulted in a large decrease in *G*′ and *G*″ compared to the sample with MNP but gelled in the absence of a magnetic field (FF + RGD + MNP sample). It should be noted that the effect of inclusion of MNP in FF hydrogels (not containing RGD) was studied previously by our group,^33^ and we found that the per se inclusion of MNP did not lead to noticeable changes in the mechanical properties, whereas the combination of MNP addition with magnetic field application during gelation resulted in strengthened anisotropic hydrogels. Anisotropy in these hydrogels appeared because of MNP migration and aggregation into column-like structures due to the attractive forces between MNP induced by the magnetic field applied during gelation. We demonstrated the anisotropic microstructure of the resulting magnetic hydrogels with light microscopy, and their anisotropic mechanical properties were demonstrated with rheological measurements under shear in two directions perpendicular to the applied field.^33^

Finally, we tested whether the hydrogels recovered their gel-like characteristics after injection through a syringe. For this purpose, we injected the samples directly onto the measuring sensor of the rheometer and subsequently monitored the evolution of the viscoelastic moduli with time. All samples showed a steady increase of *G*′ in the post-injection step (Figure 3F), although only the sample subjected to a magnetic field during this step attained values of *G*′ of the same order of magnitude as before injection, likely due to the combined effect of magnetic attraction between the particles and recovery of the supramolecular interactions between peptides. For other samples not subjected to a magnetic field during the post-injection step, the storage modulus after 120 min of self-healing was almost two orders of magnitude smaller than before injection (compare Figure 3F,C). This incomplete recovery of strength of the hydrogels after injection was also evident from the tan(δ) curves (Figure 3G). As observed, tan(δ) values were appreciably larger than 0.1 and thus typical of weak gels, especially at the shortest times and for samples not subjected to a magnetic field in the post-injection step in contrast to the values of tan(δ) ≈ 0.1 observed in gelation kinetics (Figure 3B). Although pre-injection values did not fully recover, the hydrogels tested here clearly showed self-healing behavior.

### Ex Vivo Evaluation of Hydrogels

**Figure 4:**
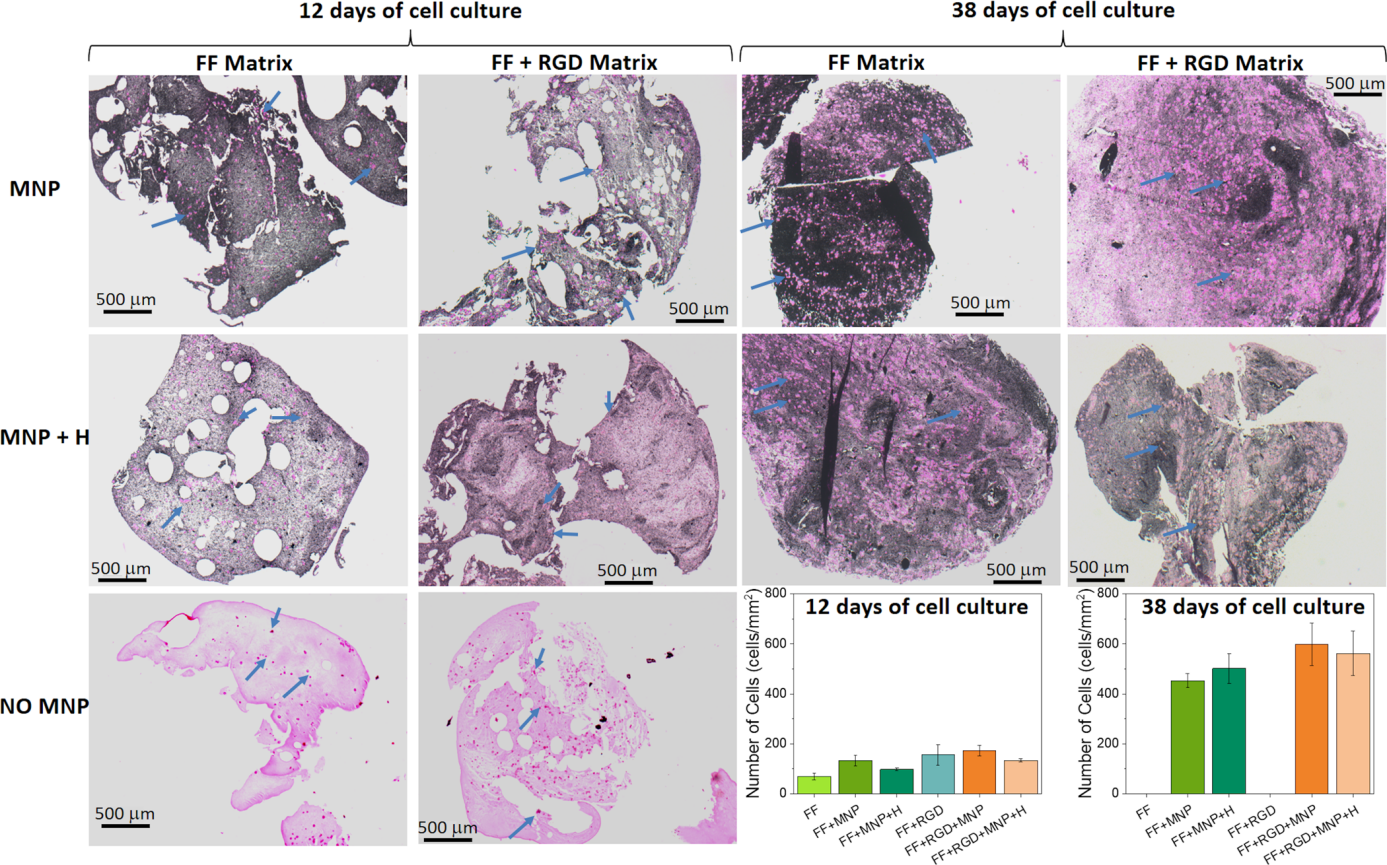
Hematoxylin and eosin staining. Bright pink dots represent cells (some of which are marked with blue arrows). Pale pink areas are the matrix media not containing magnetic nanoparticles (MNP). Matrix media containing MNP are mostly dark-colored. MNP: hydrogels containing MNP; MNP + H: hydrogels containing MNP and jellified under a magnetic field; NO MNP: hydrogels not containing MNP. FF Matrix: Fmoc-FF peptide matrix; FF + RGD Matrix: hybrid matrix based on both Fmoc-FF and Fmoc-RGD peptides. Note that hydrogels not containing MNP were completely degraded at 38 days of cell culture, thus making it impossible to perform histochemical analysis. Photographs are representative images of each experimental condition. Data in the graphs represent the mean values ± standard errors of cell count from four different cross sections from a single scaffold.

To analyze the ex vivo biocompatibility of the hydrogels, we prepared 3D cell cultures of osteoblasts in different experimental groups during two different culture periods (Figure 4). It should first be noted that samples not containing MNP were completely degraded at 38 days of cell culture and could therefore not be further evaluated at this time. Visualization with HE staining revealed a large, statistically significant (*P* < 0.05) increase in the number of cells between day 12 and day 38 (graphs in Figure 4). At both time points, the addition of RGD and/or MNP appeared to have a positive effect on cell survival/proliferation. This positive effect of RGD in cell cultures is not surprising, since it is known to be a promoter of cell adhesion.^25,47^ Regarding the role of MNP and in agreement with our results for peptide hydrogels, several previous studies reported that when MNP are added to polymeric scaffolds for tissue engineering applications, they stimulate adhesion, proliferation, and differentiation of cells in vitro and even bone formation in vivo.^48−50^ In the present work, differences between cell proliferation in different samples correlated well with differences in mechanical properties: Stronger hydrogels (higher *G*′) connected with larger numbers of cells at a given cell culture period. This result is not unexpected, given that cells are sensitive to the mechanical properties of the surrounding medium, with appropriate mechanical properties usually being considered an essential characteristic for biocompatibility of biomaterials.^51^ Therefore, our results appear to indicate that improved osteoblast proliferation was due, at least partially, to the enhanced mechanical properties of the hydrogels.

To further analyze cell proliferation in the hydrogels, we used immunohistochemical staining to investigate the potential expression of Ki-67 antigen. Note that Ki-67 antigen is a large nuclear protein (its two main isoforms have theoretical molecular weights of 345 and 395 kDa) preferentially expressed during all active phases of the cell cycle but absent in resting cells. This protein is required to maintain individual mitotic chromosomes dispersed in the cytoplasm after nuclear disassembly. Our results demonstrate that at 38 days of cell culture, 15–20% of cells were in active phases of the cell cycle for hydrogels containing MNP, regardless of whether a magnetic field was applied or whether RGD was added (Figure S2). Hydrogels not containing MNP were completely degraded at 38 days, precluding immunohistochemical analysis. Taken together, these results further support the notion that hydrogels containing MNP are suitable to promote osteoblastic cell growth.

In separate experiments, we quantified the DNA released from fibroblasts cultured in FF + RGD after 24 h, 72 h, and 1 week of cell culture. The results (Figure S3) yielded 566.2 ± 178.47 ng/μL in human cells cultured for 24 h in the presence of these hydrogels, 628.76 ± 192.95 ng/μL at 72 h, and 439.55 ± 159.41 ng/μL at 1 week. These findings confirm the presence of cells within the hydrogels after different cell culture periods and suggest that they were able to proliferate within the hydrogels. In this connection, previous work by our group demonstrated that cells remained viable and were able to proliferate within hydrogels containing MNP, as determined by the quantification of DNA and WST-1 activity.^38,52^ Future studies with longer culture periods are warranted to corroborate these preliminary results.

**Figure 5:**
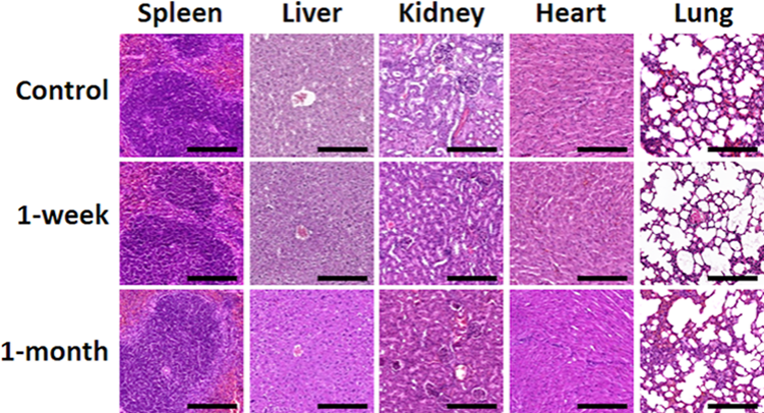
Histological analysis with hematoxylin and eosin staining of five major organs in mice (spleen, liver, kidney, heart, and lung) 1 week and 1 month after in vivo grafting. Each animal in the 1-week and 1-month groups was grafted with MNP diluted in saline, hydrogel consisting of FF + RGD, or hydrogel consisting of FF + RGD + MNP at different sites in the dorsal area, whereas controls were native nongrafted mice. Scale bar, 200 μm. Photographs are representative images of each experimental condition.

### Degradation and Swelling Behavior

**Figure 6:**
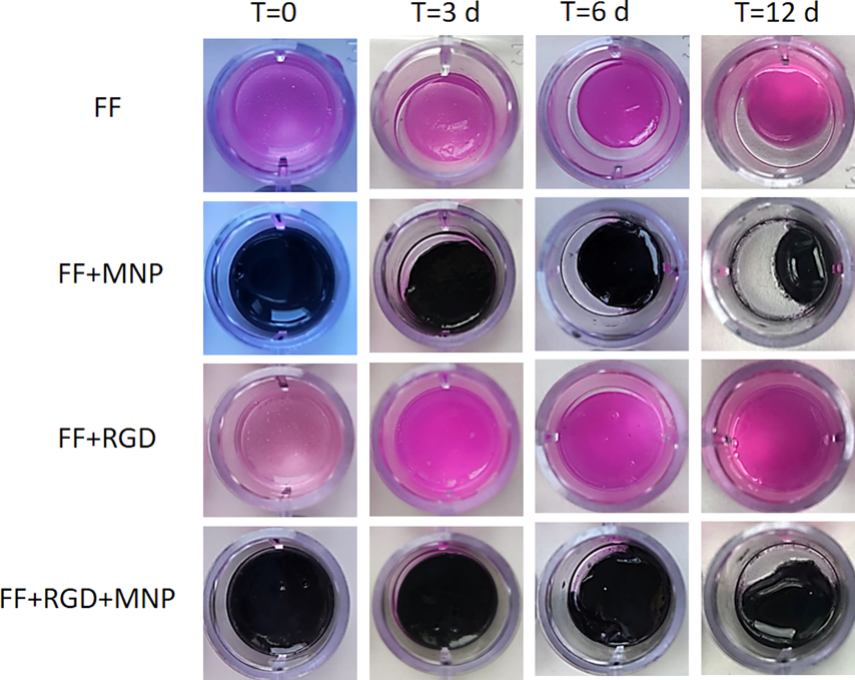
Degradation studies. Representative images of acellular hydrogels at selected periods after preparation (time, *T*, is indicated in days, d). See Figure S4 for additional images obtained at different periods after preparation.

As noted in the previous section, samples not containing MNP were completely degraded after 38 days of cell culture. To further investigate hydrogel stability, we observed the changes over time in the degradation and swelling behavior of acellular hydrogels. All samples showed signs of degradation, e.g., loss of integrity (Figure 6 and Figure S4) and decreased mass in swelling experiments (Figure S5). However, this result was not incompatible with the high stability of cellular samples as observed in experiments carried out for ex vivo evaluation, given that we found in previous work with magnetic fibrin–agarose hydrogels that the presence of cells stabilized hydrogels against degradation over a follow-up period of 30 days.^53^ In the present study, the combination of cells and MNP appeared to have a synergistic effect in preventing degradation in magnetic peptide hydrogels. A noteworthy finding was that no signs of MNP corrosion were observed, which supports the effectiveness of coating for this aim in agreement with previous work in which we reported that coating with polyethylene glycol (PEG) protected MNP from mildly acidic media.^38^

### In Vivo Biocompatibility of Particles and Hydrogels

To determine the in vivo biocompatibility of the particles and hydrogels analyzed here, they were injected subcutaneously in laboratory mice. It should be noted that magnetic hydrogels responded to a magnetic field applied after subcutaneous injection in the mice (Video S1). Mice that received implants showed no detectable signs of systemic alterations as determined by hematological testing (Table S1). Specifically, we found that all parameters for red blood cells, white blood cells, and platelets were similar in all three groups (controls, animals grafted with particles and with the hydrogels for 1 week, and animals grafted for 30 days) and none of the parameters differed significantly compared to the control animals (*P* > 0.05). We then used histological methods to analyze five major organs (spleen, liver, kidney, heart, and lung), and found that the implant was not associated with significant structural alterations in any organ after 1 week and 1 month of in vivo follow-up (Figure 5). The histological structure of the spleen was compatible with a normal organ in all cases, with abundant lymphoid cells organized in white pulp and red pulp as previously described for the normal spleen. Analysis of the liver revealed the presence of numerous hepatocytes forming normal lobules around a central vein. In the kidney, we found abundant renal corpuscles and collecting tubes with no detectable alterations. Histological analysis of the heart showed a normal structure consisting of interconnected cardiomyocytes, and the structure of the lung was also compatible with a normal organ, with abundant alveoli lined by flattened pneumocytes in all animals. None of the organs showed any microscopic signs of MNP migration or any detectable alterations. These results suggest that the particles and the different hydrogels analyzed here were safe for the host animal and were not associated with any systemic side effects.

**Figure 7:**
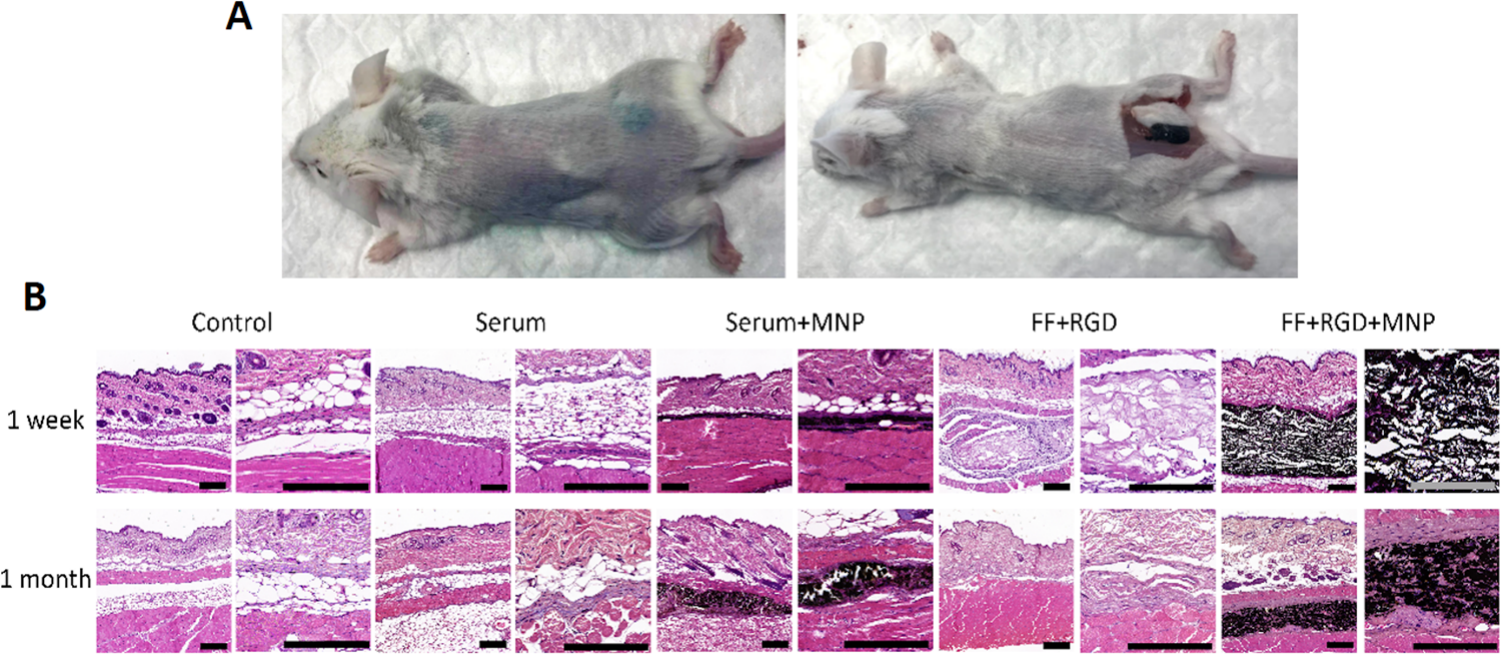
(A) Subcutaneous deposit of magnetic (FF + RGD + MNP) hydrogel 1 month after injection. (B) Histological analysis (hematoxylin and eosin staining) of the implant site in animals followed for 1 week and 1 month after in vivo grafting. Each animal in the 1-week and 1-month groups was grafted with MNP diluted in saline (Serum + MNP), hydrogel consisting of FF + RGD (FF + RGD), hydrogel consisting of FF + RGD + MNP (FF + RGD + MNP), or saline at different sites in the dorsal area, whereas controls were native non-grafted mice. For each condition, two images at different magnifications are shown. Scale bar, 200 μm. Photographs are representative images of each experimental condition.

In addition, we analyzed the implant site to search for local effects of each hydrogel. As found in hematological and histological studies of the organs, the results confirmed the biocompatibility of the particles and hydrogels and that none of the grafted animals showed any histological signs of inflammation, infection, necrosis, hemorrhage, or tumorigenesis 1 week and 1 month after in vivo grafting (Figure 7). Note also that no signs of MNP corrosion were observed in any of the implants.

Findings at the site injected with saline were compatible with normal subcutaneous tissue consisting of fibrous connective tissue and adipocytes, as seen in control non-grafted animals at both follow-up times. Implant sites in which MNP were injected diluted in saline showed a thin subcutaneous layer consisting of MNP occupying a relatively large area of subcutaneous tissue, although MNP were not seen in other neighboring structures. Interestingly, after 1 month of follow-up, the MNP were encapsulated by dense connective tissue. In the implants of FF + RGD hydrogels, we found that the injected materials were detectable at the implant site, where they formed a homogeneous fibrillar structure restricted to the injection site in the subcutaneous tissue after 1 week. However, the hydrogel was completely reabsorbed and undetectable in samples examined after 1 month. When FF + RGD + MNP hydrogels were implanted subcutaneously, we found that MNP remained concentrated at the injection site after 1 week of follow-up and did not disperse to neighbor tissues. The findings after 1 month were very similar, although we found that host cells were able to encapsulate the material implanted subcutaneously and migrate within the material.

Our preliminary morphometric analysis of control samples and tissues grafted with the different biomaterials showed no significant differences (*P* > 0.05) between groups in cell size, nuclear size, or nuclear density (Table S2). If confirmed, these results would be consistent with other evidence of the high biocompatibility of these magnetic biomaterials. In previous work, we showed that hydrogels containing MNP are highly biocompatible and do not generate adverse effects when grafted in vivo.^38,52^ Although our in vivo results strongly suggest that the novel hydrogels described in the present study are highly biocompatible, future irritation and corrosion tests should be done to further investigate the biocompatibility of these products in human skin.

Finally, we note that the results of the in vivo analyses also provide valuable information on the stability of MNP in the hydrogels and their biodistribution in animals. Stability is a key parameter related to biocompatibility, and in this regard, our results suggest that MNP remained stable within the hydrogel and did not migrate to other tissues or structures. Specifically, MNP were detected at the graft site, where they became encapsulated, but not in peripheral tissues or in distal organs. In addition, our analysis of major organs revealed no signs of MNP migration, which is consistent with the results of the blood analyses, thus suggesting that function was preserved in these major organs. Together, these results are in agreement with previous findings from our group that this type of MNP was highly stable when combined with fibrin–agarose biomaterials and showed no tendency to be released from the hydrogel to the external environment under either ex vivo or in vivo conditions.^38,52^ In particular, the present results extend our previous work^52^ on the biodistribution of MNP in Wistar rats followed for 12 weeks, in which we used a number of techniques including magnetic resonance imaging, histology, and magnetometry. These earlier results demonstrated that MNP were mostly confined to the implantation area, with some MNP biodistributed to lymphoid organs, albeit without altering their histological structure or function.

### Evaluation of Magnetic Hydrogels as Platforms for the Delivery of Cells by Injection

**Figure 8:**
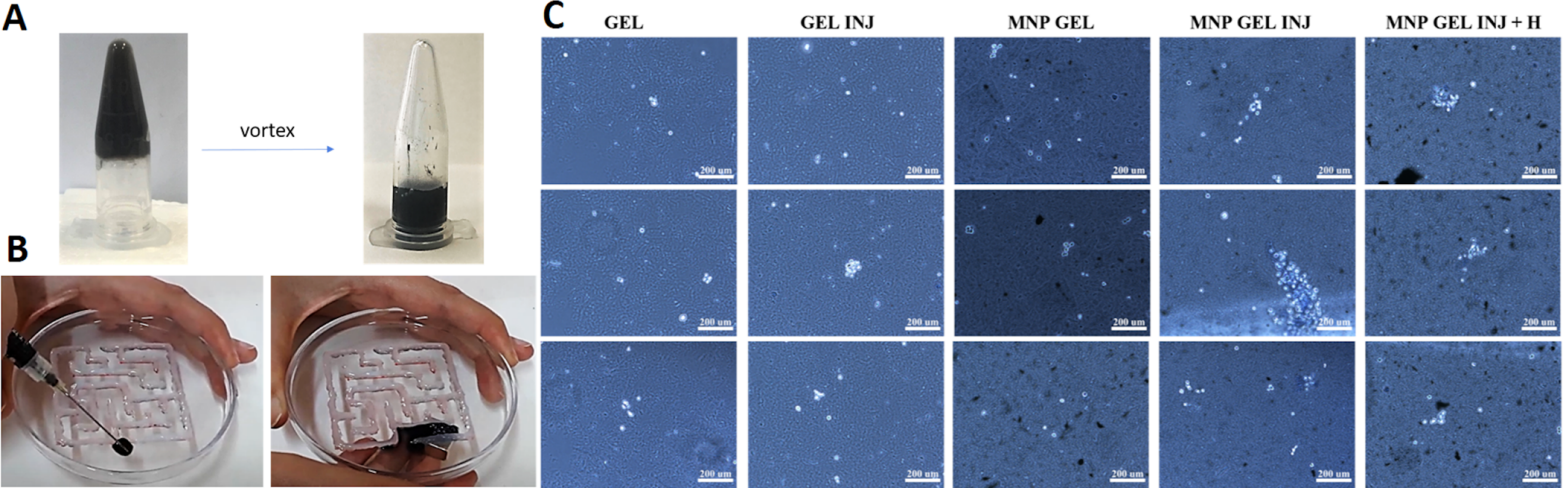
Injectability test. (A) Image of the magnetic hydrogel formed in an Eppendorf tube before and after disruption by vortex mixer. (B) Magnetic hydrogel injected and driven through a labyrinth with a magnet. (C) Trypan blue analysis of five conditions: FF + RGD hydrogel (GEL), FF + RGD hydrogel after syringe extrusion (GEL INJ), FF + RGD + MNP hydrogel (MNP GEL), FF + RGD + MNP after syringe extrusion (MNP GEL INJ), and FF + RGD + MNP after syringe extrusion and application of a magnetic field (MNP GEL INJ + H). Three photographs are shown for each condition. Cell membranes in live cells remained intact (white staining), while cell membranes were disrupted in dead cells (blue staining). Three representative images for each experimental condition are shown.

Both magnetic and nonmagnetic hydrogels retained their homogeneity after injection, without appreciable signs of phase separation (Figure 8A). Assays with magnets showed, as expected, that nonmagnetic hydrogels could not be driven by the magnet. In contrast, the noncontact magnetic force allowed controlled displacement of the magnetic hydrogels through the labyrinth without phase separation—a property that is potentially useful to achieve complete covering, filling of internal injuries, or complete cargo release and spread in a particular internal area (Figure 8B and Video S2). Previous work showed that shear-thinning hydrogels may be usable as platforms for cell delivery through a needle for potential in vivo applications.^8,10−12^ In these cases, the shear-thinning characteristic of the hydrogel, together with likely shear localization, protected most of the cells from the shear forces, which made it possible to inject them without substantial cell damage. In our experiments, we corroborated that our magnetic hydrogels could also be used for this purpose with minimal cell damage during injection, along with the additional feature of magnetic actuation by noncontact magnetic forces. As shown in Trypan blue assays (Figure 8C), most cells remained alive after the hydrogel–cell mixture was subjected to a magnetic field and subsequently to syringe extrusion.

## Conclusions

Novel short-peptide supramolecular magnetic hydrogels showing improved physical properties were prepared and studied. The inclusion of MNP in the hydrogel matrix significantly increased the robustness and stability of the hydrogels while at the same time improving injectability properties through a shear-thinning/self-healing process. These physical properties make the materials tested here ideal and versatile candidates for numerous biomedical applications. We tested these hybrid hydrogels as scaffolds for the 3D growth of osteoblasts, and our results demonstrated that these hydrogels promoted cell growth and did not degrade after 30 days of culture. The injectability of these biomaterials was evaluated in vivo by subcutaneous injections in mice. We showed that these hydrogels remain located in the area of injection without degradation 1 month after administration. During this time, hydrogels did not produce toxicity and promoted cell growth and cell migration through the hydrogel matrix. Additional properties of these hydrogels are mobilization and stiffening in response to noncontact magnetic forces. Many supramolecular short-peptide hydrogels are formed at room temperature under technically undemanding conditions and can therefore be used to trap and deliver sensitive cargos such as cells with a minimally invasive route of administration. We have shown that the magnetic hydrogels analyzed in the present study are also potential candidates for these applications. The further development of hybrid magnetic hydrogels holds considerable promise in biomedical research, particularly for in vivo applications with a minimally invasive injection strategy.

## Experimental Section

### Materials

*N*-fluorenylmethoxycarbonyl diphenylalanine (Fmoc-FF) and *N*-fluorenylmethoxycarbonyl arginylglycylaspartic acid (Fmoc-RGD) peptides were purchased from Bachem Co. (Bubendorf BL, Switzerland) and were used without further purification. Magnetic nanoparticles (MNP) made of iron (purity of 99.7% or higher) and with diameters in the range of 60–80 nm were purchased from SkySpring Nanomaterials, Inc. (Houston, TX, USA). Polyethylene glycol with a molecular mass of 400 g mol^–1^ (PEG-400), glutaraldehyde (25% solution in water), mineral oil, and sorbitan sesquioleate were all provided by Sigma-Aldrich (Burlington, MA, USA). *n*-Hexane (minimum purity of 99%) was purchased from Scharlab SL (Barcelona, Spain). Dulbecco’s modified Eagle’s Medium (DMEM), penicillin (10,000 U/mL)/streptomycin (10,000 μg/mL) solution, and UltraGlutamine 1200 mM (U-Gln) were all purchased from Lonza (Basel, Switzerland). Alpha modification of minimum essential medium (α modification with sodium bicarbonate, ribonucleosides, and deoxyribonucleosides without l-glutamine) was purchased from Sigma-Aldrich. HyClone characterized fetal bovine serum (FBS) was purchased from Cytiva (Marlborough, MA, USA).

### Functionalization of MNP

To obtain adequate integration of MNP in the peptide hydrogels, suitable functionalization was required, for which we followed a previously published protocol.^33^ Briefly, we first coated the MNP with PEG using the microemulsion method of Chatterjee et al.^54^ Then, we suspended 100 mg PEG-coated MNP in 1.5 mL of an aqueous basic (pH approx. 10.5) solution of Fmoc-FF (0.5% w/v). The resulting suspension was sonicated for 10 min and then centrifuged for 5 min at 10,000 rpm (Sigma 1-14 centrifuge) to obtain MNP with a double coating of PEG and Fmoc-FF. We removed the supernatant and used the double-coated MNP to prepare the magnetic hydrogels. A complete characterization of the physicochemical properties of these particles was reported previously.^33^ In particular, the MNP demonstrated typical soft ferromagnetic behavior with a high saturation magnetization of 1521 ± 15 kA/m, a low remnant magnetization of 62.3 ± 2.4 kA/m, and negligible effects of PEG coating on the magnetic properties.^33^

### Preparation of Hydrogels

To prepare nonmagnetic hydrogels, we proceeded as follows. First, we placed an appropriate amount of Fmoc-FF peptide in a vial, to which we added deionized water to a final concentration of 20 mM. We then immersed the resulting suspension in an ultrasonic bath (HSt Powersonic 603) to achieve homogeneous dispersion of the peptides (approximately 1 h). Then, we added small amounts of a 0.5 M NaOH solution by pipette until a clear solution was obtained at a pH of approximately 10.5. We measured the pH value with a HACH sensION+ pH 3 pH meter that was previously calibrated with pH 4, 7, and 10 buffer solutions. Then, we added appropriate amounts of Fmoc-RGD powders to deionized water in a pressure vial to obtain a peptide concentration of 20 mM. This solution became transparent immediately with a pH of approx. 3.98. To prepare hybrid Fmoc-FF/Fmoc-RGD solutions, we mixed them at a ratio of 7:3, whereas for pure Fmoc-FF hydrogels (without Fmoc-RGD peptide), we mixed the Fmoc-FF peptide solution with water at a ratio of 7:3. Finally, we induced gelation in the hybrid Fmoc-FF/Fmoc-RGD or pure Fmoc-FF peptide solutions by adding equal volumes of a 50:50 mixture of DMEM and water. The final pH of the Fmoc-FF hydrogel was approximately 7.4, and the final pH of the Fmoc-FF/Fmoc-RGD hydrogel was approximately 7.1.

To prepare magnetic hydrogels, we slightly modified the previous protocol by adding appropriate amounts of MNP with double-coating (PEG + Fmoc-FF) to the peptide solutions before the addition of DMEM. The final concentration of MNP was 0.1 vol %. In some cases, we induced the formation of anisotropic, elongated structures of MNP in the hydrogel microstructure by applying a vertical magnetic field (*H* = 15 kA m^–1^) during the first hour of gelation after the addition of DMEM. A solenoid connected to a DC power supply was used to apply the magnetic field.

All hydrogels were prepared according to the procedure detailed above. In all cases, hydrogel properties were characterized 24 h after DMEM was added to allow for complete gelation. A summary of the different hydrogels studied in this work is shown in Table 1.

**Table 1:** Summary of the Main Preparation Characteristics of Hydrogels Prepared for the Present Work

sample	peptides^a^used for the matrix	addition of MNP^b^	application of magnetic field^c^
FF	Fmoc-FF	no	no
FF + MNP	Fmoc-FF	yes	no
FF + MNP + H	Fmoc-FF	yes	yes
FF + RGD	Fmoc-FF + Fmoc-RGD^d^	no	no
FF + RGD + MNP	Fmoc-FF + Fmoc-RGD^d^	yes	no
FF + RGD + MNP + H	Fmoc-FF + Fmoc-RGD^d^	yes	yes

a Peptide concentration was 20 mM in all cases.

b Concentration of MNP was 0.1 vol %. MNP were previously double-coated with PEG + Fmoc-FF.

c Strength of the magnetic field was *H* = 15 kA m^–1^ and was maintained during the first hour of gelation.

d The Fmoc-FF-to-Fmoc-RGD ratio was 7:3.

### Physicochemical Characterization of Hydrogels

#### Fourier-Transform Infrared Spectroscopy

We recorded FTIR spectra with a PerkinElmer Two FTIR ATR spectrometer. The nonmagnetic and magnetic hydrogels were compressed onto the diamond crystal, and the spectra were scanned over a range of 4000 to 450 cm^–1^.

#### Transmission Electron Microscopy (TEM)

The hydrogels were examined with a LIBRA 120 PLUS Carl Zeiss apparatus. Hydrogels were vortexed and diluted with water 1:10. Hydrogel samples were placed on a 300-mesh copper grid stained with 1% aqueous uranyl acetate solution and dried at room temperature for 30 min. At least six different areas of hydrogel for each experimental condition were analyzed.

#### Circular Dichroism

The CD spectra were recorded with a Jasco J-815 spectropolarimeter and 150 W xenon lamp. The hydrogels were jellified into a 0.1 mm quartz cell (Hellma 0.1 mm quartz Suprasil) according to the protocol described above. Spectra were obtained from 200 to 320 nm with a 1-nm step and 0.5 s integration time per step at 20 °C.

### Mechanical Evaluation of Hydrogels

#### Gelation Kinetics

We investigated the gelation kinetics of hydrogels by obtaining rheological measurements with a Haake MARS III controlled-stress rheometer (Thermo Fisher Scientific, Waltham, MA, USA) equipped with a double cone–plate sensor 60 mm in diameter at a 2° apex angle (sensor DC60/2° Ti L). For these assays, we followed the protocol for the preparation of magnetic and nonmagnetic hydrogels described above and poured the mixture in the measuring system of the rheometer before adding the water/DMEM mixture. We then added the water/DMEM mixture directly to the peptide mixture in the measuring system of the rheometer to induce gelation and immediately afterward subjected the gelling sample to an oscillatory shear strain of fixed frequency (1 Hz) and amplitude (γ_0_ = 0.001) while monitoring the resulting viscoelastic moduli as a function of time. For FF + RGD + MNP + H samples, we applied a 15 kA/m magnetic field during gelation by using a coil connected to a power supply placed coaxially to the rheometer axis. The strain amplitude (γ_0_ = 0.001) used in these assays was low enough to ensure that formation of the gel microstructure was unperturbed. Characterization was carried out at a constant temperature of 37.0 ± 0.1 °C. We obtained measurements for at least three fresh samples for each experimental condition.

#### Characterization of the Mechanical Properties of Hydrogels

We characterized the mechanical properties of the hydrogels under oscillatory shear strains by using the same rheometer as in the previous section, equipped with a double-plate sensor of 35 mm in diameter and rough surfaces to avoid wall slip (sensor P35 Ti L S serrated, Thermo Fisher Scientific). Characterization was carried out at a constant temperature of 37.0 ± 0.1 °C. First, we subjected the hydrogels to amplitude sweeps, for which the frequency of oscillation was kept at 1 Hz and the amplitude of the oscillatory strain, γ_0_, was increased stepwise from 0.0001 to 2 along a logarithmic ramp. These measurements provided the values of storage (*G*′) and loss (*G*″) moduli as a function of γ_0_. Then, we performed frequency sweep tests, for which the amplitude of the shear strain was fixed at γ_0_ = 0.001 and the frequency of oscillation was increased stepwise from 0.1 to 16 Hz. These measurements provided the values of *G*′ and *G*″ of the hydrogels as a function of frequency (i.e., the mechanical spectra).

A fresh sample was used for each amplitude and frequency sweep and each experimental condition, and we obtained measurements for at least three different samples for each experimental condition. In this article, we report the corresponding mean values and standard errors of the measurements.

#### Self-Healing Behavior of Hydrogels

We investigated the self-healing behavior of the hydrogels of the present work by obtaining rheological measurements with the same rheometer equipped with a double cone-plate sensor (sensor DC60/2° Ti L) as described above. The hydrogel was produced in a vial according to the gel preparation protocol noted above and was maintained for 24 h. Then, it was disrupted with a vortex mixer, drawn into a syringe with a 20G gauge needle, and injected into the measuring system of the rheometer. The combination of vortex mixing and the stress produced during injection led to the complete disruption of the sample. The sample was then immediately subjected to an oscillatory shear strain of fixed frequency (1 Hz) and amplitude (γ_0_ = 0.001) while monitoring the resulting viscoelastic moduli as a function of time. For FF + RGD + MNP + H samples, we applied a 15 kA/m magnetic field during gelation with a coil connected to a power supply placed coaxially to the axis of the rheometer. We obtained measurements for at least three fresh samples for each experimental condition.

#### Ex Vivo Biocompatibility of Hydrogels

The gel preparation protocol reported above was slightly modified to incorporate cells in the hydrogels. First, we sterilized all reagents and carried out gel preparation under sterile conditions. Then, we followed the previous steps for gel preparation, except for the last step: Instead of DMEM, we added normal human osteoblasts (hOB #35, passage 8) dispersed in complete αMEM (composition as below). We chose a density of 1 million cells per culture well (human primary osteoblasts, Lonza, Basel, Switzerland). We added αMEM supplemented with Hyclone FBS 10%, U-Gln (2 mM), and penicillin/streptomycin (100 U/mL and 100 μg/mL, respectively) and cultured the samples under standard culture conditions for two different culture periods of 12 and 38 days. At the end of each period, we examined the samples after hematoxylin and eosin (HE) staining (samples were fixed for 72 h in formaldehyde at room temperature and then paraffin-embedded; sections 4 μm thick were made, deparaffinized in xylene, hydrated, and stained with HE). Cell proliferation for selected samples was investigated after immunostaining with an anti-human Ki-67 antigen antibody (monoclonal mouse anti-human Ki-67 antigen, clone MIB-1, from Agilent, Santa Clara, CA, USA).

In separate experiments, we cultured human fibroblasts (20,000 cells in 1 mL of hydrogel per culture well) in FF + RGD samples for 24 h, 72 h, and 1 week under standard culture conditions and used a NanoDrop 2000 UV–Vis spectrophotometer (Thermo Fisher Scientific) to quantify the DNA released according to previously established protocols.^38^ We tested at least three different hydrogel samples per experimental condition.

### Degradation and Swelling Behavior

For degradation studies, we deposited the hydrogels (three samples per experimental condition) in well plates, which were then immersed in DMEM and kept at 37 °C in a laboratory incubator. Every 24 h, the DMEM medium was changed, and we monitored the physical integrity of the hydrogels by direct observation and photography without artificial magnification for a total period of 12 days.

To study swelling behavior, we prepared the hydrogels (four samples of 500 μL per each experimental condition), placed them in Eppendorf tubes, added 500 μL of water, and stored the tubes at room temperature. Every 24 h, we removed the supernatant and calculated the mass of the hydrogels by subtracting the mass of the empty tube from the mass of the tube + swelled hydrogel. Mass measurements were obtained with a microbalance. Then, we added 500 μL of water and stored the hydrogels at room temperature until the next measurement.

#### In Vivo Biocompatibility of Particles and Hydrogels

Biocompatibility of the MNP and hydrogels was assessed in 12 BALB/c laboratory mice. The animals were deeply anesthetized with ketamine and acepromazine, and a subcutaneous injection was made in each mouse (see Video S1) consisting of 300 μL of (i) MNP diluted in saline (0.1% v/v PEG-Fmoc-FF-coated MNP), (ii) hydrogel consisting of FF + RGD (FF/RGD at a ratio of 7:3), (iii) hydrogel consisting of FF + RGD + MNP (0.1% v/v PEG-Fmoc-FF-coated MNP in a hydrogel with FF/RGD at a ratio of 7:3), or (iv) saline only (used as a control). Each animal received one injection of each of the four materials in different parts of the dorsal area, with a separation of 1 cm between implant sites. Six animals were killed by intraperitoneal injection of a euthanasia solution (Eutanax 200, Fatro Ibérica, Barcelona, Spain) after 7 or 30 days of follow-up, and the main organs (spleen, liver, kidney, heart, and lung), along with the four injection areas, were removed and analyzed histologically. As a control, we used one BALB/c mouse with no implants for each of the two follow-up periods.

#### Evaluation of Magnetic Hydrogels as Platforms for the Delivery of Cells by Injection

First, we investigated the injectability of the hydrogels and their potential actuation by magnetic forces. We prepared the hydrogel in an Eppendorf tube, and after complete gelation, we disrupted the hydrogel with a vortex mixer. Immediately thereafter, we drew the hydrogel into a syringe with a 0.3 mm gauge and injected it into a petri dish to which we had previously glued a homemade labyrinth. Then, with the help of a permanent magnet, we tried to move the injected hydrogel through the labyrinth. In a separate experiment, we proceeded as described, but before the disrupted hydrogel was drawn into the syringe, we mixed it with human fibroblasts (200,000 cells per mL of hydrogel). In some experimental groups, we subjected the cell/hydrogel mixture to a magnetic field in a petri dish as described above. In other groups, we injected the cell/hydrogel mixture into culture wells, and after 10 min, we analyzed cell viability by Trypan blue staining to observe whether the hydrogel protected cells from mechanical damage caused by shearing forces and field-induced displacement of the magnetic particles. As a control, we analyzed cell/hydrogel samples that were not subjected to syringe use or to a magnetic field. These experiments were done for three different hydrogels in each experimental condition. For cell viability studies, we used at least three samples for each experimental condition.

#### Statistical Analyses

For blood analyses, morphological analysis of histological results, and other statistical comparisons, the data obtained for each study group were compared with the Mann–Whitney statistical test in RealStatistics software (Dr. Charles Zaiontz, Purdue University, West Lafayette, IN, USA). In all cases, *P* values below 0.05 were considered statistically significant.

#### Ethics Statement

This study was approved by the Ethics Committees of the Junta de Andalucía (Spain) within the framework of research project FIS2017-85954-R (MCIN/AEI/10.13039/501100011033/FEDER, Spain). The ex vivo studies with human cells were authorized by the Servicio Andaluz de Salud (Consejería de Salud, Junta de Andalucía). The in vivo characterization was performed in compliance with European Union and Spanish Government guidelines for the ethical care of animals (EU Directive no. 63/2010, RD 53/2013). In vivo experiments were authorized by the Dirección General de la Producción Agrícola y Ganadera (approval number 18/12/2017/167, Consejería de Agricultura, Pesca y Desarrollo Rural, Junta de Andalucía).

## Supporting Information Available

(Figure S1) CD and FTIR of Fmoc-FF and Fmoc-FF/Fmoc-RGD samples, (Figure S2) immunohistochemical staining for Ki-67 antigen expression after 38 days of cell culture in samples containing MNP, (Figure S3) DNA released from fibroblasts cultured in FF + RGD hydrogels, (Figure S4) degradation studies of acellular hydrogels, (Figure S5) swelling behavior of acellular hydrogels, (Table S1) average values of main hematological parameters, (Table S2) morphometric analysis of implantation sites in mice grafted with different biomaterials (PDF)
(Video S1) Subcutaneous injection of hydrogel in a mouse followed by responsiveness under the influence of a magnet (MP4)
(Video S2) Injection of magnetic hydrogel and movement through a labyrinth by noncontact magnetic force (MP4)
