# Supplementary material for: Injectable Magnetic-Responsive Short-Peptide Supramolecular Hydrogels: Ex Vivo and In Vivo Evaluation
Source: ACS Appl Mater Interfaces. 2021 Oct 14;13(42):49692–704. doi: 10.1021/acsami.1c13972 (PMC8554763; doi:10.1021/acsami.1c13972)
Supplement: Supplementary file 1 — am1c13972_si_001.pdf [file am1c13972_si_001.pdf]

SUPPLEMENTARY INFORMATION

Injectable magnetic-responsive short-peptide supramolecular hydrogels: ex vivo and in vivo evaluation

Mari C. Mañas-Torres,[1,3] *Cristina Gila-Vilchez,*[2,3] *Francisco Vazquez-Perez,*[2] *Pavel Kuzhir,*[4] *David Momier,*[5] *Jean-Claude Scimeca,*[5] *Arnaud Borderie,*[6] *Marianne Goracci,*[6] *Fanny Burel-Vandenbos,*[6] *Cristina Blanco-Elices,*[3,7] *Ismael Rodríguez,*[7,8] *Miguel Alaminos,*[3,7,*] *Luis Álvarez de Cienfuegos,*[1,3,*] *Modesto T. Lopez-Lopez*[2,3,*]

[1] Universidad de Granada, Departamento de Química Orgánica, Facultad de Ciencias, Unidad de Excelencia de Química Aplicada a Biomedicina y Medioambiente, 18071 Granada, Spain

[2] Universidad de Granada, Departamento de Física Aplicada, Facultad de Ciencias, 18071 Granada, Spain

[3] Instituto de Investigación Biosanitaria ibs.GRANADA, Granada, Spain

[4] Université Côte d'Azur, CNRS UMR 7010, Institute of Physics of Nice, Parc Valrose, 06108 Nice, France

[5] Université Côte d'Azur, CNRS UMR 7277, Institute of Biology Valrose, 06107 Nice, France

[6] Université Côte d'Azur, Department of Pathology, CHU Nice, 06107 Nice, France

[7] Department of Histology and Tissue Engineering Group, Faculty of Medicine, University of Granada, 18071 Granada, Spain

[8] Department of Histology, Faculty of Dentistry, National University of Cordoba, Cordoba, Argentina

* Corresponding authors: malaminos@ugr.es (M. Alaminos), lac@ugr.es (L. Álvarez de Cienfuegos), modesto@ugr.es (M.T. Lopez-Lopez)

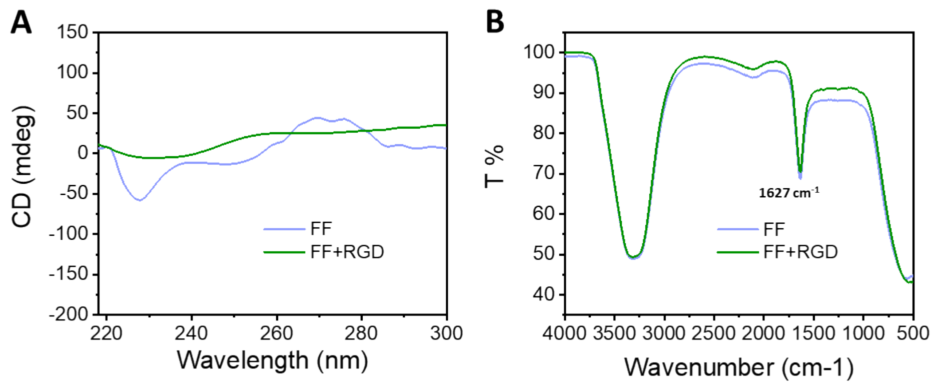

Figure S1. Circular dichroism (CD) and Fourier-transform infrared spectroscopy (FTIR) spectra of the hydrogels. A) CD of Fmoc-FF and Fmoc-FF/Fmoc-RGD; B) FTIR of Fmoc-FF and Fmoc-FF/Fmoc-RGD.

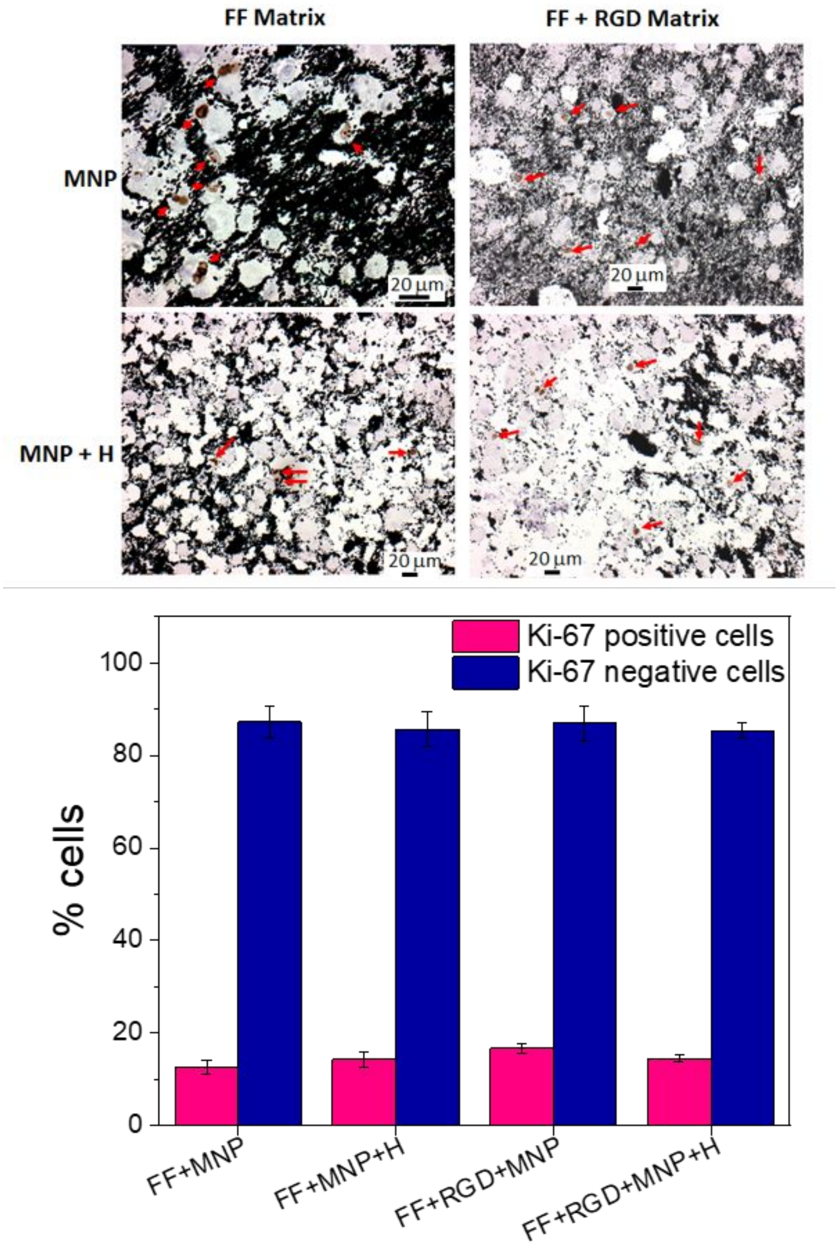

Figure S2. Immunohistochemical staining for Ki-67 antigen expression after 38 days of cell culture in samples containing MNP. Brown nuclear staining is observed in cells in active phases of the cell cycle (some cells marked with red arrows), whereas blue nuclear staining identifies cells in the resting phase of the cell cycle. Because sections were 3 µm thick, some cells appear with no nucleus. **MNP**: Hydrogels containing MNP; **MNP + H**: Hydrogels containing MNP and jellified under a magnetic field; **FF Matrix**: Fmoc-FF peptide matrix; **FF + RGD Matrix**: hybrid matrix based on both Fmoc-FF and RGD peptides. Note that hydrogels containing no MNP were completely degraded after 38 days of cell culture, so immunohistochemical analysis was not possible. Photographs are representative images of each experimental condition. Data in the graphs represent the

mean values ± standard errors of cell counts from three different cross-sections from the same scaffold.

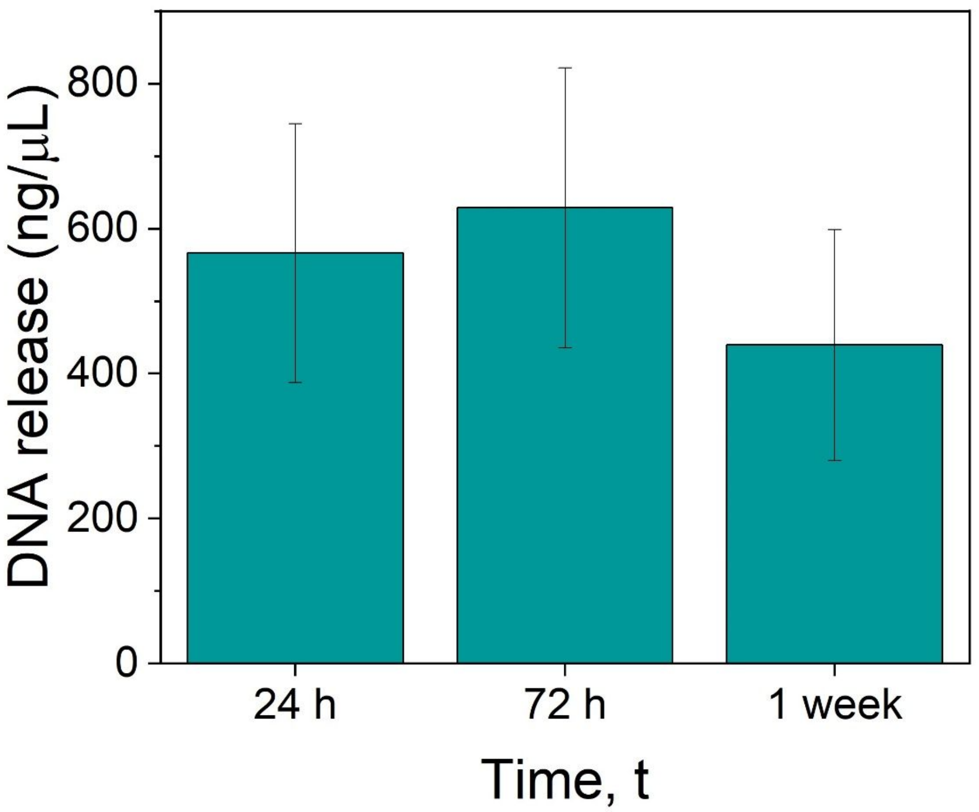

Figure S3. DNA released from fibroblasts cultured in FF+RGD hydrogels. Data represent mean values ± standard deviations for at least three different repeats and three different hydrogels (nine values) per experimental condition.

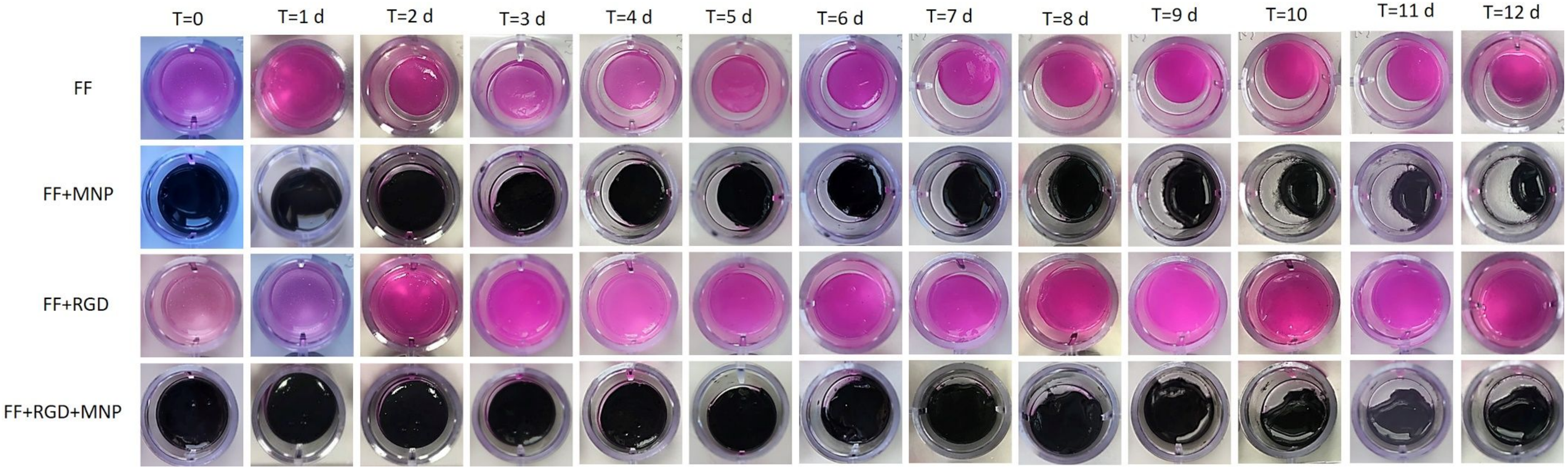

Figure S4. Degradation studies. Representative images of acellular hydrogels on different days after preparation (time, T, is indicated in days, d).

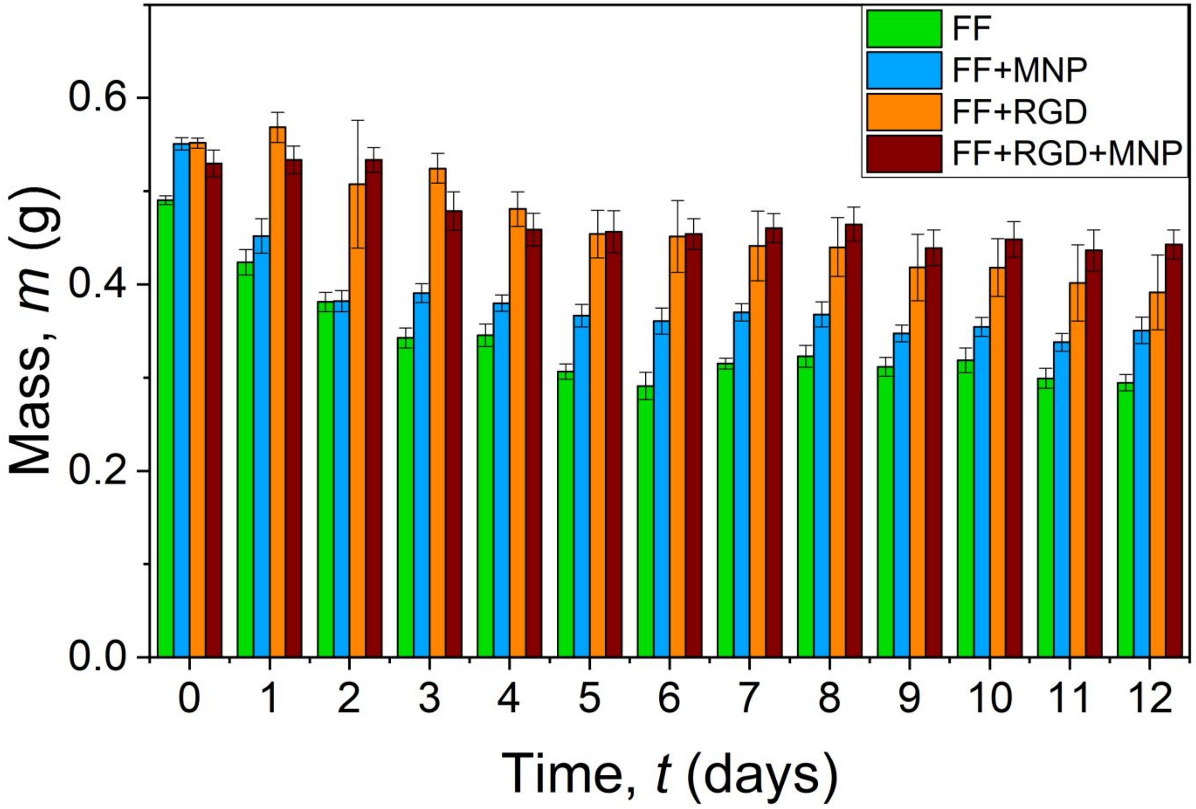

Figure S5. Swelling behavior. Mass of hydrogels (after removing the water supernatant) as a function of time. Data represent mean values ± standard errors for four different samples per experimental condition.

Table S1. Average values of the main hematological parameters in blood from animals in the control (CTR) group (native mice) and animals grafted with the different materials after 1 week (1W) and 30 days (1M). Note that each animal in the experimental groups received an implant of each of the four materials 1 cm apart in different parts of the dorsal area.

	**RBC**	**HGB**	**HCT**	**MCV**	**MCH**	**MCHC**	**RDW_SD**	**RDW_CV**	**WBC**	**LYM#**	**MXD#**	**NEUT#**	**PLT**	**PDW**	**MPV**	**P_LCR**
CTR	7915000 ±845000	12.4 ±1.2	40.05 ±4.15	50.65 ±0.15	15.65 ±0.15	31 ±0.2	27.9 ±1.1	13.8 ±2	3300 ±1200	2750 ±1050	200 ±0	350 ±150	44500 ±9500	14 ±0.3	8.15 ±0.65	21.75 ±5.35
1W	8740000 ±411290.65	13.2 ±1.69	44.37 ±2.21	50.77 ±0.88	15.05 ±1.33	29.68 ±2.72	28.13 ±0.4	14.32 ±0.61	3816.67 ±1851.94	3150 ±1650.15	433.33 ±258.2	233.33 ±81.65	42833.33 ±20855.85	11.35 ±5.46	9.88 ±2.31	37.03 ±19.9
1M	8138333.33 ±449329.13	12.53 ±0.83	40.7 ±2.4	50 ±0.55	15.4 ±0.3	30.8 ±0.89	27.42 ±0.12	12.65 ±0.16	3900 ±616.44	3250 ±484.77	350 ±137.84	300 ±126.49	44333.33 ±23114.21	10.52 ±5.29	7.53 ±1.1	16.2 ±10.16

Abbreviations:

RBC: red blood cells

HGB: hemoglobin

HCT: hematocrit

MCV: mean corpuscular volume

MCH: mean corpuscular hemoglobin

MCHC: mean corpuscular hemoglobin concentration

RDW_SD: red blood cell distribution width and standard deviation

RDW_CV: red blood cell distribution width and coefficient of variation

WBC: white blood cells

LYM#: lymphocyte count

MXD#: mixed cell count

NEUT#: neutrophil count

PLT: platelet count

PDW: platelet distribution width

MPV: mean platelet volume

P_LCR: platelet/large cell ratio

Table S2. Morphometric analysis of implantation sites in mice grafted with the different biomaterials evaluated in the present study. In each study group, cells found at the graft site were quantified to determine cell size, nuclear size and cell density (number of cells per 2500 μm^2 surface area). Values are shown as the average ± standard deviation. 1W: results after 1 week of *in vivo* follow-up; 1M: results at 1 month (30 days) of *in vivo* follow-up.

	CONTROL - 1W	CONTROL - 1M	SERUM - 1W	SERUM - 1M	SERUM+ MNP - 1W	SERUM+ MNP - 1M	FF+RGD – 1W	FF+RGD - 1M	FF+RGD+ MNP -1W	FF+RGD+ MNP - 1M
Cell size	48.2±6.8	47.85±0.64	49.1±6.8	48.2±5.36	48.35±8.87	47.78±17.24	48.3±11.23	48.53±13.99	48.27±3.52	48.95±7.42
Nuclear size	20.8±4.33	20.67±3.76	20.73±1.12	20.64±2.84	20.87±4.31	20.53±3.32	20.4±4.03	20.03±3.36	20.28±3.92	20.53±2.04
Cellular density	10.33±1.53	10.67±2.52	10.33±1.53	10.2±3.19	10.5±5.32	10.6±3.51	10.5±3.54	10.4±4.39	10.6±2.7	10.5±1.73
